# Actual Situation of Thromboembolic Prophylaxis in Obesity Surgery: Data of Quality Assurance in Bariatric Surgery in Germany

**DOI:** 10.1155/2012/209052

**Published:** 2012-07-09

**Authors:** Christine Stroh, D. Luderer, R. Weiner, T. Horbach, K. Ludwig, F. Benedix, Stefanie Wolff, C. Knoll, H. Lippert, T. Manger

**Affiliations:** ^1^Department of General, Abdominal and Pediatric Surgery, SRH Hospital, Straße des Friedens 122, 07548 Gera, Germany; ^2^Institute for Quality Assurance in Operative Medicine, Otto-von-Guericke University, 39106 Magdeburg, Germany; ^3^Municipal Hospital Sachsenhausen, Frankfurt/Main, Germany; ^4^Friedrich-Alexander University, 91054 Erlangen-Nuremberg, Germany; ^5^Municipal Hospital, Schwabach, Germany; ^6^Municipal Hospital, Rostock Suedstadt, Germany; ^7^Otto-von-Guericke Universität Magdeburg, 39106 Magdeburg, Germany; ^8^StatConsult, 39112 Magdeburg, Germany

## Abstract

*Background*. Evidence-based data on optimal approach for prophylaxis of deep venous thrombosis (VTE) and pulmonary embolism (PE) in bariatric operations is discussed. Using antithrombotic prophylaxis weight adjusted the risk of VTE and its complications have to be balanced with the increased bleeding risk. *Methods*. Since 2005 the current situation for bariatric surgery has been examined by quality assurance study in Germany. As a prospective multicenter observational study, data on the type, regimen, and time course of VTE prophylaxis were documented. The incidences of clinically diagnosed VTE or PE were derived during the in-hospital course and follow up. *Results*. Overall, 11,835 bariatric procedures were performed between January 2005 and December 2010. Most performed procedures were 2730 gastric banding (GB); 4901 Roux-en-Y-gastric bypass (RYGBP) procedures, and 3026 sleeve gastrectomies (SG). Study collective includes 72.5% (mean BMI 48.1 kg/m^2^) female and 27.5% (mean BMI 50.5 kg/m^2^) male patients. Incidence of VTE was 0.06% and of PE 0.08%. *Conclusion*. VTE prophylaxis regimen depends on BMI and the type of procedure. Despite the low incidence of VTE and PE there is a lack of evidence. Therefore, prospective randomized studies are necessary to determine the optimal VTE prophylaxis for bariatric surgical patients.

## 1. Introduction

Obesity has developed into an epidemic. Approximately 1.7 billion people are overweight, and 312 million are obese [1, 2]. In Germany in 2009, 60.1% of male and 42.9% of female population were overweight [3]. Morbid obesity is associated with increased health costs and an increased mortality risk [4, 5]. 

As a result of new technologies with lower risks and better long-term results, bariatric and metabolic surgeries have grown in popularity in recent years. The number of operations performed is rapidly increasing. However, bariatric surgery is associated with numerous peri- and postoperative complications. Venous thromboembolism is the most common postoperative complication. Obesity needs to be considered as one of the most serious factors predisposing patients to the development of thrombosis and pulmonary embolism. In addition, the risk for thromboembolism in metabolic surgery is high, and its detection is complicated. The guidelines of the German Society of Surgery suggest that there is a high risk of deep venous thrombosis (VTE) and pulmonary embolism (PE) for patients who undergo bariatric surgery. An even higher risk for the development of a thrombosis is reported by the German AWMFguidelines [6]. International guidelines refer to an independent risk for the occurrence of fulminate pulmonary embolism in obese patients. Current “ASBMS” guidelines recommend local compressions and anticoagulation [7].

Actually, the optimal approach for prophylaxis of VTE in bariatric operations as well as obese patients is unknown. The risk of VTE and its complications has to be balanced with the increased bleeding risk. In the literature, there have been no randomized studies examining the correlation between VTE, pulmonary embolism (PE), and the various options for VTE prophylaxis in bariatric surgery [8]. Recent published meta-analysis show the relatively low risk with standard regimen for antithrombotic prophylaxis, but an increasing bleeding risk, when weight-adjusted dose of heparin were used [9].

The aim of this study evaluating the data of the German Nationwide Survey on Bariatric Surgery was to investigate the specific complications of thrombosis and pulmonary embolism in the perioperative course in Germany and to compare our findings with data from the literature.

## 2. Patients and Methods

Data from the Study for quality assurance in bariatric surgery in Germany have been prospectively obtained from January 1, 2005 [10] using an online data registry. For current evaluation, data from 2005 to 2010 were analyzed. Incidence and risk of VTE for the most performed metabolic procedures gastric banding (GB), sleeve gastrectomy (SG), and Roux-en-Y-gastric bypass (RYGBP) have been evaluated. Peri-interventional characteristics and parameters, such as BMI as well as the frequency, type, application mode, and duration of medication in VTE and PE, were determined in connection to the operative procedure. Follow-up data, which are available for 63.8% of patients, were used to elucidate the incidence of VTE and PE after discharge. To appropriately interpret the results, a literature search was initiated.

## 3. Results

### 3.1. Operations

Since 2005 the number of primary metabolic operations has been increasing, rapidly. 

We reporting on 12,  296 primary bariatric procedures including 668 implantations of gastric balloons (BIB), 2730 GB, 4901 RYGBP, 3042 SG, 120 biliopancreatic diversion (BPD), and 163 duodenal switch (DS) and 211 other procedures. 

### 3.2. Demographic Data

In total 72.5% female patients and 27.5% male patients were operated between 2005 and 2010. Mean age for all patients was 42.2 years (range 11–79 years) and mean BMI 48.8 kg/m² (range 23.7–115 kg/m²).

Mean age for women (40.9 years, range 11–79 years) was significantly lower than for men (43.6 years, range 14–79 years) (*P* > 0.001). Mean BMI was for women 48.1 kg/m² (range 23.7–115.0 kg/m²) and 50.1 kg/m² (range 27.4–10.2 kg/m²) for men. 

### 3.3. Comorbidities

For risk analysis the incidence of comorbidities is important. In German Nationwide Survey on Bariatric Surgery the incidence of comorbidities is high. At the different periods from 2005 to 2010 the incidence on comorbidities did not change. 57.3% of patients suffering on hypertension, 17.5% on pulmonary diseases, 9.7% on cardiac comorbidities, 14.9% on gastroesophageal reflux disease, and 43.6% on skeleton disease. Incidence on patients with insulin-dependent diabetes is 10.0% and with noninsulin-dependent diabetes is 19.1% (*P* < 0.001) (Table 1).

### 3.4. Operation and Thromboembolic Prophylaxis

Prophylaxis of VTE and PE depends on the kind of operation. Through the observation period from 2005 to 2010, a significant reduction of patients undergoing GB procedure and who had previously undergone prophylaxis for thromboembolism with medication was found (*P* < 0.001), respectively (Table 2). In cases of complex surgical interventions like biliopancreatic diversion (BPD) and duodenal switch (DS), prophylactic medication was given to all patients.

The duration of prophylaxis of thromboembolism correlated significantly with the length of hospital stay, which ranged from 6 to 8 days (*P* < 0.001) (Table 3).

LMWH was used for prophylaxis in 98.5% of the patients; only in 1.5% of patients were unfractionated heparin used for prophylaxis. No prophylaxis with cava filters is reported to German Nationwide Survey.

In 2005, there was no association between BMI and the frequency of prophylaxis administration for thromboembolism. In contrast, there was a tendency to administer low-molecular-weight heparin twice a day during the following years. Patients who received two administrations showed a higher BMI (once a day, BMI = 48.5 kg/m²; twice a day, BMI = 51.5 kg/m² (*P* < 0.001). Therefore, patients with two applications for thromboembolism prophylaxis showed a significantly higher BMI through the whole study period (Table 4).

### 3.5. Risks for VTE and PE

VTE risk and risk of fatal PE could not evaluate any significant difference in dependence on operative procedure for the most performed procedures GB, SG, and RYGBP (Table 5).

Overall incidence of VTE was 0.06% (8/11835) and for PE 0.08% (10/11835). Comparing gender-specific risks for VTE we found no significance difference between men and women. Risk for female patients is 0.06% and for male patients 0.09% (*P* = 0.5216). Increasing BMI correlates with a higher incidence on VTE until BMI of 49.9 kg/m². In patients with BMI above 50 kg/m² the VTE incidence dropped down (Table 6). Evaluated risk for VTE at the 90 first postoperative days is for all procedures ≤0.10%.

Incidence of PE for GB, SG, and RYGBP in German Nationwide Survey was ≤0.20% (Table 5). According gender-specific aspects exist no significance difference for developing PE 90 days postoperatively (*P* = 0.5085). Risk factor for female patients was 0.08% (relative risk 0. 8773%) and for male patients 0.12% (relative risk 1.3242%) (Table 6).

## 4. Discussion

German Nationwide Survey has evaluated the risk of VTE (0.06%) and PE (0.08%) in bariatric surgery Germany between 2005 and 2010. VTE or PE has been reported to the survey, when it was diagnosed by vascular ultrasound, CT scan, or echocardiography. 

According to the literature, the risk for the development of a PE after bariatric surgery is between 0.1% and 1.3% [11, 12]. The risk for thromboembolic complications in bariatric surgery in patients who did not receive in-hospital chemoprophylaxis is 2.4% [12, 13]. In particular, fatal PE (0.3%) needs to be considered as the strongest independent factor for perioperative mortality. DeMaria et al. have reported a mortality of 0.23% due to PE (10/4431 patients) [14]. Data of BOLD-Registry have shown a VTE risk 90 days postoperatively of 0.42% (*n* = 73, 971). 73% of VTE in this study have been developed after patients discharge during the first 30 days [15]. However, no evidence-based data on optimal prophylaxis exist [9, 14]. According German AWMF-Guidelines and ACCP-Guidelines visceral surgery and morbid obesity is associated with the highest risk for VTE and PE [16]. Benefit and risk of bleeding has to be considered if weight-adjusted LWMH is used for prophylaxis [9, 14, 15]. Using LWMH prophylaxis with pneumatic compression is widely used in bariatric surgery. Advantage of pneumatic compression in laparoscopic bariatric surgery is defending of venous stasis in lower extremity. 

German Nationwide Survey has a significant reduction of antithrombotic prophylaxis for patients undergoing GB evaluated. This fact can be associated with the fact that GB is implanted during a short hospital stay of one or two days in several hospitals. For these patients we did not evaluate a higher incidence of VTE. As a bias of our study we cannot exclude that clinical symptoms of VTE or PE for these patients were not documented at the registry.

In patients with BMI above 50 kg/m² the incidence of VTE dropped down. In this fact as a bias of the study we cannot exclude that patients with BMI above 50 kg/m² were treated with higher dose of LMWH, mostly. A second limitation is that patients with BMI above 55–60 kg/m² in Germany were first treated with BIB to reduce overall complication rate. Implantation of BIB is most performed without VTE prophylaxis. For laparoscopic interventions, data obtained in a randomized study by Nguyen et al. suggest that hypercoagulability caused a higher incidence of PE [17]. In addition, pneumoperitoneum is a potential risk factor due to the resulting venous stasis of the lower extremity, which is increased by the anti-Trendelenburg position [18]. In German Nationwide Survey, however, this effect could not be detected for RYGBP as well as for SG or GB.

According to the data on German Nationwide Survey in BOLD-Registry there was no influence of operation on the incidence of VTE. BOLD Registry shows an incidence of VTE after GB of 0.16% and after BPD of 5.56%. Risk for VTE was higher if operation was performed at laparoscopic approach (1.53% versus 0.34%) [15]. 

In German Nationwide Survey in patients with BMI of 45 kg/m² to 49.9 kg/m² the highest risk of VTE and PE was observed. Patients developing PE were in average 4.9 years older, had on average an 3.9 kg/m² higher BMI, and had a higher incidence of VTE in medical history 16.5% versus 3.7%. The gender-specific aspect of VTE and PE was not detected in German Nationwide Survey. Data of Winegar et al. show a higher risk for male patient (HR 2.32, 95% CI 1.81–2.98) [15].The problem of BOLD Registry is missing informations on medication, dose of medication, and peri- and postoperative treatment. 

Scholten et al. have shown the positive effect of a certain dosage of Enoxaparin on the risk for PE. Higher doses resulted in a significantly lower rate of PE. A higher incidence of bleeding was not estimated [19]. Data contrary to the association of dosage and BMI have been obtained in a randomized study by Kalfarentzos et al., who did not observe any difference in the frequency of thromboembolic episodes under various dosages of Fraxiparin [20]. Data of German Nationwide Survey did not evaluate a higher incidence of bleeding when using a higher dosage of LWMH. At the most participating centers, a higher dosage is used only in high BMI patients without using exactly weight-adjusted LMWH. Further studies favor the administration of unfractionated-heparin depending on the monitoring of factors Xa in high-risk patients [21–23]. However, the associations between the anti-Factor-Xa level, dosage of LWMH, and occurrence of bleeding have been excluded in a previous study [24]. 

Evidence-based data on the optimal duration; type and dosage of LWMH for prophylaxis of VTE and PE do not exist in the literature.

Cohort analysis of 17, 434 patients after bariatric operations describe an outpatient department VTE incidence of 0.88%. More than 74% of thromboembolic complications occur after patients discharge and one-third after 1 month postoperatively. Highest incidence of VTE and PE was observed after RYGBP (OR = 0.31). Evaluated risk factors are male gender (OR = 1.68), age above 55 years (OR = 2.18), nicotine abuse (OR = 1.86), and medical history of VTE (OR = 7.48) [25]. Data of “PROBE-Study” had evaluated as risk factors for the development of a PE after bariatric surgery, smoking, age >40 years, BMI >60 kg/m², and previous history of venous thromboembolism [11]. 

For high-risk patients with a BMI >50 kg/m², a medical history of VTE, surgical interventions at the pelvis, and heart failure in further studies, the positive effect of an IVC filter was found [26–29]. 

Actual data on PE and VTE in bariatric surgery show similar to evaluated data of German Nationwide Survey on bariatric surgery a low incidence of 0.49% (*n* = 29, 323) [9, 15, 30]. The current ASMBS statement recommends performing compression therapy and medication for the prophylaxis of thromboembolism. The AWMF guidelines classify patients with a BMI >30 kg/m² as high risk, but there is a lack of appropriate recommendations for the prophylaxis of thromboembolism in bariatric surgery [6, 9].

## 5. Conclusions and Recommendations

Present published review on thromboembolism after laparoscopic bariatric surgery has shown that available evidence on use of antithrombotic prophylaxis is limited [9].

Based on a literature search, the following predictive factors were evaluated for the occurrence of thromboembolism after bariatric interventions:

1. age above 50 years,
2. smoking,
3. thrombosis/pulmonary embolism in medical history [31, 32],
4. venous stasis [16],
5. BMI > 50 kg/m²,
6. visceral obesity,
7. pulmonary hypoventilation [13],
8. ventricle septum defect (VSD) and aortic stenosis [33].

The dosage of NMH should be adapted for risk patients with

-  BMI > 50 kg/m² [34],
-  age above 50 years,
-  male gender,
-  venous insufficiency,
-  hypoventilation syndrome,
-  smoking, and
-  thrombosis in the medical history.

However, evidence-based data on the recommendations mentioned above are lacking. Therefore, randomized or observational studies with large number of cases are required to optimize medication and mechanical and interventional prophylaxis for thromboembolism [9, 16, 35, 36].

## Figures and Tables

**Table 1:** Incidence of comorbidities.

	Total	
	(*n*)	(%)
Hypertension	7042	57.3
Skeleton disease	5366	43.6
Sleep apnoea	2380	19.4
Noninsulin dependent diabetes	2347	19.1
Pulmonary disease	2153	17.5
Without	2001	16.3
GERD	1836	14.9
Cardiac disease	1193	9.7
Nicotin abuse	275	2.2
LE in medical history	108	0.9

**Table 2:** VTE prophylaxis in dependence on operation.

Year	2005 (%)	2006 (%)	2007 (%)	2008 (%)	2009 (%)	2010 (%)	Total (%)
BIB	54.2	59.5	23.6	21.9	28.6	32.7	36.7
GB	100	99.2	96.9	98.5	97.7	95.6	97.9
SG			97.7	99.2	96.1	99.4	98.8
RYGBP	100	100	99.3	99.8	97.0	99.2	99.2
BPD	100	100	100	100	100	100	100
DS	100	100	100	100	100	100	100

**Table 3:** Duration on VTE prophylaxis in days.

Year	2005	2006	2007	2008	2009	2010
Mean length of prophylaxis in days (d)	6.86 (1–47)	7.62 (1–52)	7.16 (1–86)	8.20 (1–210)	9.07 (1–110)	9.15 (1–105)
Median of prophylaxis in days (d)	7	7	6	6	6	6

**Table 4:** Correlation between BMI and VTE prophylaxis.

BMI (kg/m^²^)				Year		
	2005	2006	2007	2008	2009	2010	Total
Applications per day							
Once daily	48.5	48.3	48.1	48.4	48.4	48.4	48.4
Twice daily	50.0	55.0	51.5	51.6	51.0	51.6	51.5
*P*	0.179	<0.001	<0.001	<0.001	<0.001	<0.001	<0.001

**Table 5:** Diagnosed events of VTE and PE during the first 90 postoperative days in dependence on operative procedure.

Procedure	Number of patients (*n*)	Number of VTE (*n*)	VTE risk (95% confidence interval) (%)	Number of patients (*n*)	PE risk (95% confidence interval) (%)
SG	3026	3	0.10 (0.02–0.29)	6	0.20 (0.07–0.43)
RYGBP	4901	3	0.06 (0.01–0.18)	3	0.06 (0.01–0.18)
GB	2730	2	0.07 (0.01–0.26)	1	0.04 (0.00–0.20)

**Table 6:** Diagnosed events of VTE and PE during the first 90 postoperative days in dependence on gender and BMI.

	VTE risk (95% confidence interval) (%)	VTE relative risk (95% confidence interval) (%)	*P*	PE risk (95% confidence interval) (%)	PE relative risk (95% confidence interval) (%)	*P*
Male	0.09 (0.02–0.27)	1.3691 (0.5594–3.3509)	0.5085	0.12 (0.03–0.31)	1.3242 (0.6056–2.8953)	0.5216
Female	0.06 (0.02–0.14)	0.8608 (0.5032–1.4725)		0.08 (0.03–0.17)	0.8773 (0.5611–1.3715)	

BMI (kg/m²)						
30.0–34.9	0.00 (—)			0.00 (—)		
35.0–39.9	0.00 (—)			0.00 (—)		
40.0–44.9	0.07 (0.01–0.26)	1.129	0.3090	0.04 (0.00–0.20)	0.342 (0.040–2.929)	0.7783
45.0–49.9	0.11 (0.02–0.32)	1.682		0.18 (0.06–0.42)	1.697 (0.491–5.866)	
>50.0	0.06 (0.01–0.19)	1.000		0.11 (0.03–0.25)	1.000	

## Abbreviations

BIB: Intragastric balloon
BPD: Biliopancreatic diversion
GB: Gastric banding
DS: Duodenal switch
NMH: Heparin of low molecular weight
PE: Pulmonary embolism
RYGBP: Roux-en-Y gastric bypass
SG: Sleeve gastrectomy
VTE: Deep venous thrombosis.
